# HIV prevalence and high-risk sexual behaviours among MSM repeat and first-time testers in China: implications for HIV prevention

**DOI:** 10.7448/IAS.17.1.18848

**Published:** 2014-07-02

**Authors:** Xue Bai, Jie Xu, Jie Yang, Bo Yang, Maohe Yu, Yongjun Gao, Willa M Dong, Zunyou Wu

**Affiliations:** 1 School of Public Health, Anhui Medical University, Hefei, China; 2 National Centre for AIDS/STD Control and Prevention, Chinese Centre for Disease Control and Prevention, Beijing, China; 3 Tianjin Dark Blue Working Group, Tianjin, China; 4 Tianjin Center for Disease Control and Prevention, Tianjin, China; 5 Tianjin Hongqiao District Center for Disease Control and Prevention, Tianjin, China

**Keywords:** MSM, repeat testing, VCT, HIV, risk behaviour, China

## Abstract

**Introduction:**

Little is known about HIV testing, HIV infection and sexual behaviour among bathhouse patrons in China. This study aims to assess differences in HIV prevalence and high-risk sexual behaviours between repeat and first-time testers among men who have sex with men (MSM) attending bathhouse in Tianjin, China.

**Methods:**

Between March 2011 and September 2012, a HIV voluntary counselling and testing station was established in a gay bathhouse, which provided HIV testing and conducted a survey among participants recruited through snowball sampling. Differences in demographic and high-risk sexual behaviours between repeat and first-time testers were assessed using the chi-square test. Univariate and multivariate logistic regression analyses were conducted to identify predictors for HIV infection.

**Results:**

Of the 1642 respondents, 699 (42.6%) were repeat testers and 943 (57.4%) were first-time testers. Among repeat testers, a higher proportion were men aged 18 to 25, single, better educated, had a history of STIs and worked as male sex workers or “money boys” (MBs). Repeat testers were less likely to report having unprotected anal intercourse in the past six months. The overall HIV prevalence was 12.4% (203/1642). There was no difference in HIV prevalence between repeat (11.2%, 78/699) and first-time (13.3%, 125/943) testers. The HIV prevalence increased with age among first-time testers ($\chi_{trend}^{2}=9.816$, *p*=0.002). First-time MB testers had the highest HIV prevalence of 34.5%.

**Conclusions:**

MSM attending bathhouse had an alarmingly high HIV infection rate, particularly in MB. Targeted interventions are urgently needed especially focusing on older MSM and MBs.

## Introduction

Sexual transmission has become the primary route of HIV transmission in China, and men who have sex with men (MSM) constitute almost one-third of all cases of new HIV infections [1]. The proportion of newly reported HIV cases attributable to homosexual transmission rose from 2.5% in 2006 to 13.7% in 2011 [1]. Among MSM, HIV prevalence is estimated to be between 2.5 and 7.4% and increasing at an annual rate of 1.1% [2, 3].

Voluntary counselling and testing (VCT) is an important strategy for the prevention and control of HIV and AIDS by engaging clients in risk reduction. A recent model of the epidemic in China found that a four-fold increase in VCT would avert 42,000 cases of HIV and 11,000 HIV-related deaths over the next five years [4]. Since 2007, significant resources have been directed towards scaling up MSM testing services under China’s National Plan for HIV/AIDS Prevention and Control [1]. In the period after the adoption of the plan, there were significant increases in both lifetime testing rates (from 24 to 47%) and rates of testing in the past 12 months (from 21 to 38%) among MSM [5]. In 2009, the lifetime rates of testing reached 72% in Beijing among younger MSM, suggesting that repeat testing is increasing among this group [6].

Previous research has found that repeat testers and first-time testers differ in several ways. Among MSM, repeat testers are often younger, better educated and unmarried compared to first-time testers [7–9]. However, repeat testing is associated with several risk factors for HIV: a higher number of sexual partners [10], having ever had an STI [11], unprotected anal intercourse (UAI) [12, 13] and working as a male sex worker [14]. Additionally, in a study among MSM in seven US cities, over 75% of repeat testers who seroconverted did so within a year after their last test [13]. Continued engagement in risk behaviour by this specific group of repeat testers may undermine the effectiveness of VCT among MSM [15]. There has not yet been a study examining differences in HIV risk between MSM repeat and first-time testers in China, despite the distinct demographic and risk behaviour characteristics of these two groups [6–9, 14]. Therefore, in the context of increasing rates of testing, it is necessary to understand whether repeat testing may reduce the risk of HIV among MSM. The objective of this study was to assess the prevalence of sexual risk behaviours and HIV infection of repeat and first-time MSM testers attending a bathhouse VCT station.

## Methods

### Study participants and setting

This study was conducted in the city of Tianjin, which is a major Chinese city located 137 km east of Beijing. The overall HIV prevalence among MSM in Tianjin was 6.4% in 2008 [16].

In March 2011, an HIV VCT station was established in a gay bathhouse by the National Centre for AIDS/STD Control and Prevention (NCAIDS) of the Chinese Center for Disease Control and Prevention (Chinese CDC), Tianjin CDC, Tianjin Hongqiao District CDC and the Tianjin Dark Blue Working Group, a non-governmental organization (NGO) for MSM. The cost of entry to the bathhouse was 20 CNY (approximately US$3.28). Between March 2011 and September 2012, 1642 men attending this bathhouse were recruited through both snowball sampling and convenient sampling. Volunteers from the Tianjin Dark Blue Working Group encouraged the patrons to seek testing, and the patrons who accepted voluntary testing then referred their friends to receive HIV testing. Eligibility criteria included being 18 years of age or older, having had oral intercourse or AI with another male in the last 12 months, and being willing and able to provide informed consent. Attendees who self-reported being diagnosed with HIV were excluded. After obtaining informed consent, participants completed rapid HIV testing and an anonymous face-to-face questionnaire administered by trained staff from the Tianjin Dark Blue Working Group.

### Study design

The questionnaire collected information on socio-demographics, AIDS knowledge, sexual risk behaviours, HIV risk awareness and HIV testing history. Socio-demographics included date of birth, marital status, residence, ethnicity, length of stay in Tianjin, educational level, STI history and money boy (MB) status. Participants reported whether or not they had been diagnosed with STIs in the past year. MBs who sell sex to men were defined as those who self-disclosed their status during the questionnaire survey. Additionally, participants were assessed on their knowledge of HIV and AIDS prevention and transmission through eight questions adapted from the Chinese national surveillance survey. The eight questions included the following: (1) whether people can look healthy when infected with HIV; (2) whether mosquito bites can transmit HIV; (3) whether HIV can be transmitted through sharing meals; (4) whether HIV can transmit through blood transfusion and using blood products; (5) whether the use of non-sterile needles can transmit HIV; (6) whether HIV can be spread from mother to child; (7) whether condom use can reduce HIV transmission; and (8) whether having sex with only one person will prevent HIV infection. Participants were considered knowledgeable about HIV and AIDS if six or more questions for each area were answered correctly. HIV risk awareness was defined as the perception of the likelihood of being infected with HIV, and was divided into five levels of very high, high, moderate, low and none. For HIV testing history, participants were considered first-time testers if they had never received HIV testing before and repeat testers if they had ever received HIV testing and were not diagnosed with HIV.

### HIV testing and counselling

All participants were presented to pre-testing HIV counselling and then testing. It took half an hour to receive the results, during which the informed consent and the questionnaire were completed. HIV testing was performed initially using a rapid saliva test (AWARE, Beijing, China) for screening. If the initial result was negative, the subject was considered HIV negative and was given a post-test counselling following national guideline. If the initial screening test was positive, then an immediate repeated rapid saliva HIV test with the same brand of test kit and a rapid HIV blood test (ACON, Hangzhou, Zhejiang province, China) would be performed. If the results from either or both tests were positive or indeterminate, the participant was considered HIV positive and was referred to treatment services. NGO staff who received extensive VCT training provided pre-test counselling and post-test counselling in a routine manner as a risk reduction strategy.

### Statistical analysis

Data were double entered into a database constructed using EpiData 3.0 (EpiData Association, Odense, Denmark). Age was recoded categorically for analysis. Analyses of differences in the demographics and behavioural characteristics between first-time and repeat testers were conducted using the chi-square test. To identify predictors for HIV infection, we conducted univariate and multivariate logistic regression analyses among the overall MSM group, first-time tester and repeat tester groups, respectively. All variables with *p<*0.1 in the univariate logistic regression model were then entered into a multivariate regression model using forward stepwise selection with the significance level set at *p*=0.05. Statistical analysis was performed using SPSS version 13.0 software (IBM Corporation, Chicago, IL).

### Ethic statement

The study protocol has been reviewed and approved by the Institutional Review Board of NCAIDS of Chinese CDC (IRB approval number: X130205267). A written informed consent was obtained from all study participants. Incentives of 50 CNY in cash (equivalent to US$8.19) and a pack of three condoms as a gift were provided for subjects who participated in the study.

## Results

### Study participants

All participants who were approached and received testing between March 2011 and September 2012 consented to participating in the study. The mean age of the 1642 MSM who completed the survey was 36 years (with a range of 18 to 76 years). Most were aged 35 to 44 years (33.7%), followed by men aged 25 to 34 years (33.6%), older than 45 years (19.3%) and younger than 25 years (13.4%). Most were married (57.4%), ethnically Han (97.4%) and did not have a Tianjin household registration (56.2%). However, the majority had resided in Tianjin for more than two years (62.3%). Roughly three quarters of the participants had completed middle school education. In the past year, 75 participants (4.6%) were diagnosed with STIs and 88 (5.4%) worked as MBs.

### Demographic characteristics of repeat and first-time testers

Of the 1642 respondents, 699 men (42.6%) had received HIV test once or more in the past and 943 men (57.4%) were first-time testers. Among first-time testers, a higher proportion consisted of men older than 35 years (58.2%) compared to repeat testers (45.9%) (*χ* ^2^=24.428, *p*<0.001). A higher percentage of first-time testers were married or cohabitating with a female partner (62.3%) than repeat testers (50.8%) (*χ* ^2^=21.971, *p*<0.001). Though the majority of first-time testers had attended at least some high school (71.5%), repeat testers had a slightly higher proportion of those with this level of education (77.4%) (*χ* ^2^=7.317, *p*=0.007).

Among repeat testers, the proportion of participants who had STIs in the past year were 6.4%, which was significantly higher than that of first-time testers (3.2%) (*χ* ^2^=9.766, *p*=0.002). The percentage of respondents who worked as sex workers was 8.4% and 3.1% in repeat testers and first-time testers, respectively, which differed significantly (*χ* ^2^=22.784, *p*<0.001) (Table 1).

**Table 1:** Demographic characteristics of repeat and first-time testers in MSM attending bathhouse in Tianjin, China

Variable	Total	Repeat testers	First-time testers	χ^2^	p

		*n=*699	*n=*943		
Age (years)				24.428	<0.001
<25	220 (13.4)	109 (15.6)	111 (11.8)		
25–34	552 (33.6)	269 (38.5)	283 (30.0)		
35–44	553 (33.7)	203 (29.0)	350 (37.1)		
≥45	317 (19.3)	118 (16.9)	199 (21.1)		
Marital status				21.971	<0.001
Unmarried/divorced or widowed	699 (42.6)	344 (49.2)	355 (37.6)		
Married/cohabitated	943 (57.4)	355 (50.8)	588 (62.3)		
Residence				0.427	0.514
Tianjin	720 (43.8)	313 (44.8)	407 (43.2)		
Other province	922 (56.2)	386 (55.2)	536 (56.8)		
Ethnicity				0.001	0.97
Han Chinese	1600 (97.4)	681 (97.4)	919 (97.5)		
Other	42 (2.6)	18 (2.6)	24 (2.5)		
Length of stay in Tianjin				0.762	0.683
Less than 3 months	457 (27.8)	191 (27.3)	266 (28.2)		
Between 3 months and 2 years	162 (9.9)	74 (10.6)	88 (9.3)		
More than 2 years	1023 (62.3)	434 (62.1)	589 (62.5)		
Educational level				7.317	0.007
Completed middle school	427 (26.0)	158 (22.6)	269 (28.5)		
High school or above	1216 (74.0)	541 (77.4)	674 (71.5)		
Had STIs in the past year				9.766	0.002
Yes	75 (4.6)	45 (6.4)	30 (3.2)		
No	1567 (95.4)	654 (93.6)	913 (96.8)		
Male sex worker (MB)				22.784	<0.001
Yes	88 (5.4)	59 (8.4)	29 (3.1)		
No	1554 (94.6)	640 (91.6)	914 (96.9)		

### Differences in HIV and AIDS knowledge and high-risk sexual behaviours between repeat and first-time testers

Compared with first-time testers, repeat testers reported a higher awareness rate of HIV and AIDS knowledge (95.1% vs. 88.2%; *χ* ^2^=23.787, *p*<0.001), and were significantly more likely to practice AI (91.1% vs. 84.7%; *χ* ^2^=14.989, *p*<0.001) and commercial male sex (13.0% vs. 7.6%; *χ* ^2^=13.011, *p*<0.001). A majority of both repeat testers (64.2%) and first-time testers (88.0%) reported having engaged in UAI with men and this difference was statistically significant (*χ* ^2^=33.216, *p*<0.001). First-time testers were also more likely to ever practice UAI during group sex (73.0% vs. 57.0%; *χ* ^2^=5.260, *p*=0.022), have UAI with their last male commercial sex partner (36.1% vs. 19.8%; *χ* ^2^=5.440, *p*=0.02) and unprotected vaginal sex with a female partner within the past six months (87.5% vs. 74.9%; *χ* ^2^=14.627, *p*<0.001) (Table 2).

**Table 2:** HIV and AIDS knowledge and high-risk sexual behaviours among repeat testers and first-time testers attending bathhouse in Tianjin, China

	Repeat testers		First-time testers			
				
Variable	*N*	*n* (%)	*N*	*n* (%)	*χ* ^2^	*p*
AIDS knowledge	699	665 (95.1)	943	832 (88.2)	23.787	<0.001
HIV risk awareness	699		943		11.755	0.003
High (reference)		39 (5.6)		31 (3.3)		
Moderate		232 (33.2)		379 (40.2)		
Low or none		428 (61.2)		533 (56.5)		
Had anal intercourse(AI)	699	637 (91.1)	943	799 (84.7)	14.989	<0.001
Frequency of AI in last week	637		799		0.179	0.673
≤2		501 (78.6)		621 (77.7)		
>2		136 (21.4)		178 (22.3)		
UAI with recent male sex partner	637	224 (35.2)	799	421 (52.7)	44.002	<0.001
UAI in the past 6 months	637	409 (64.2)	799	623 (88.0)	33.216	<0.001
Had group sex	699	86 (12.3)	943	100 (10.6)	1.153	0.283
UAI in group sex	86	49 (57.0)	100	73 (73.0)	5.26	0.022
Had AI with commercial male sex partner(s)	699	91 (13.0)	943	72 (7.6)	13.011	<0.001
UAI during last commercial male sex	91	18 (19.8)	72	26 (36.1)	5.44	0.020
UAI during commercial male sex (past 6 months)	91	38 (41.8)	72	37 (51.4)	1.501	0.221
Had sex with any female partner(s)	699	219 (31.3)	943	336 (35.6)	3.318	0.069
Unprotected sexual intercourse with last female partner	219	164 (74.9)	336	294 (87.5)	14.627	<0.001
Unprotected sexual intercourse with any female partner (past 6 months)	219	143 (65.3)	335	262 (78.2)	11.229	0.001

### HIV prevalence and associated risk factors among overall MSM population, repeat and first-time testers

A total of 203 MSM were tested HIV positive with an overall prevalence of 12.4% (203/1642). Seventy-eight men among the 699 repeat testers (11.2%) and 125 of the 943 first-time testers (13.3%) were found to be positive for HIV. There was no significant difference in HIV prevalence between first-time testers and repeat testers (*χ* ^2^=1.629, *p*=0.202) (Table 3).

**Table 3:** HIV prevalence and risk factors among MSM attending bathhouse in Tianjin, China

		Univariate		Multivariate	
			
Variable	HIV prevalence	OR (95% CI)	*p*	OR (95% CI)	*p*
Had HIV testing					
Yes	11.2 (78/699)	1.0			
No	13.3 (125/943)	1.2 (0.9–1.6)	0.202		
Age (years)					
<25	10.5 (23/220)	1.0		1.0	
25	9.8 (54/552)	0.9 (0.6–1.6)	0.779	1.2 (0.7–2.0)	0.530
35	13.6 (75/553)	1.3 (0.8–2.2)	0.243	2.2 (1.3–3.8)	0.005
>45	16.1 (51/317)	1.6 (1.0–2.8)	0.064	2.9 (1.6–5.3)	<0.001
Marital status					
Unmarried/divorced or widowed	12.7 (89/699)	1.0			
Married/cohabitated	12.1 (114/943)	0.9 (0.7–1.3)	0.695		
Residence					
Tianjin	10.1 (73/720)	1.0		1.0	
Other province	14.1 (130/922)	1.5 (1.1–2.0)	0.016	1.6 (1.1–2.2)	0.007
Ethnicity					
Han Chinese	12.4 (198/1600)	1.0			
Other	11.9 (5/42)	1.0 (0.4–2.5)	0.927		
Length of stay in Tianjin					
Less than 3 months	14.4 (66/457)	1.0			
Between 3 months and 2 years	9.9 (16/162)	0.6 (0.4–1.2)	0.143		
More than 2 years	11.8 (121/1023)	0.8 (0.6–1.1)	0.163		
Educational Level					
Completed middle school	15.2 (65/427)	1.0			
Some high school or above	11.4 (138/1215)	0.7 (0.5–1.0)	0.038		
Had STIs in the past year					
Yes	21.3 (16/75)	2.0 (1.1–3.6)	0.018	2.1 (1.1–3.7)	0.008
No	11.9 (187/1567)	1.0		1.0	
Male sex worker (or money boy “MB”)					
Yes	28.4 (25/88)	3.1 (1.9–5.0)	<0.001	4.4 (2.6–7.7)	<0.001
No	11.5 (178/1554)	1.0		1.0	
AIDS knowledge					
Knowledgeable	12.3 (184/1497)	1.0			
Not knowledgeable	13.1 (19/145)	1.1 (0.6–1.8)	0.777		
HIV risk awareness					
High	12.9 (9/70)	1.0			
Moderate	11.8 (72/611)	0.9 (0.4–1.9)	0.793		
Low or none	12.7 (122/961)	1.0 (0.5–2.0)	0.969		
Had anal intercourse (AI)					
Yes	12.5 (179/1436)	1.0			
No	11.7 (24/206)	0.9 (0.6–1.5)	0.740		
Number of AI in last week					
≤2	11.8 (157/1328)	1.0			
>2	14.6 (46/314)	1.3 (0.9–1.8)	0.172		
UAI with recent male sex partner					
Yes	13.8 (89/645)	1.0			
No	11.4 (114/997)	0.8 (0.6–1.1)	0.156		
UAI in the past 6 months					
Yes	13.2 (136/1032)	1.0			
No	11.0 (67/610)	0.9 (0.8–1.1)	0.192		
Had group sex					
Yes	14.5 (27/186)	1.0			
No	12.1 (176/1456)	0.8 (0.5–1.3)	0.344		
UAI in group sex					
Yes	19.7 (24/122)	1.0			
No	11.8 (179/1520)	0.7 (0.6–0.9)	0.012		
Had AI with commercial male sex partner(s)					
Yes	12.9 (21/163)	1.0			
No	12.3 (182/1479)	0.9 (0.6–1.5)	0.832		
UAI in the last commercial male sex					
Yes	15.9 (7/44)	1.0			
No	12.3 (196/1598)	0.7 (0.3–1.7)	0.470		
UAI in commercial male sex (past 6 months)					
Yes	16.0 (12/75)	1.0			
No	12.2 (191/1567)	0.9 (0.6–1.2)	0.329		
Had sex with any female partner(s)					
Yes	11.4 (63/555)	1.0			
No	12.9 (140/1087)	1.2 (0.8–1.6)	0.374		
Unprotected sexual intercourse with last female partner					
Yes	12.3 (50/405)	1.0			
No	12.4 (153/1237)	1.0 (0.7–1.4)	0.990		
Unprotected sexual intercourse with any female partner (past 6 months)					
Yes	12.0 (55/458)	1.0			
No	12.5 (148/1184)	1.0 (0.9–1.2)	0.786		

In the overall univariate analysis of the MSM group (Table 3), non-local residents (OR=1.5, 95% CI=1.1–2.0, *p*=0.016) were found to be significantly associated with an increased likelihood of being HIV infected. MSM having an STI diagnosis within the past year (OR=1.4, 95% CI=1.0–1.9, *p*=0.038) and MB (OR=3.1, 95% CI=1.9–5.0, *p*<0.001) were more likely to be HIV positive. Protective factors included educational level above middle school (OR=0.7, 95% CI=0.5–1.0, *p*=0.038) and not having UAI during group sex (OR=0.7, 95% CI=0.6–0.9, *p*=0.012).

In the overall multivariate analysis of the MSM group (Table 3), men older than 35 years (aOR=2.2, 95% CI=1.3–3.8, *p*=0.005) and men older than 45 years (aOR=2.9, 95% CI=1.6–5.3, *p*<0.001) were more likely to be infected with HIV than men younger than 25. MSM without a local residence remained at an increased likelihood of being HIV infected (aOR=1.6, 95% CI=1.1–2.2, *p*=0.007). For those having had an STI in the past year, the likelihood of HIV infection was two times higher (aOR=2.1, 95% CI=1.1–3.7, *p*=0.008). Compared with non-sex workers, MBs (aOR=4.4, 95% CI=2.6–7.7, *p*<0.001) were 4.4 times to be infected with HIV.

Among repeat testers, in the univariate analysis (Table 4), non-local residents were two times more likely to be HIV positive (OR=2.0, 95% CI=1.2–3.2, *p*=0.009). Having lived in Tianjin for more than two years were less likely to be infected with HIV (OR=0.5, 95% CI=0.3–0.9, *p*=0.012). Working as an MB also significantly increased the likelihood for HIV infection (OR=3.1, 95% CI=1.6–5.9, *p*=0.001). In the multivariate analysis, likelihood of infection was associated with non-local residents (aOR=1.7, 95% CI=1.0–2.9, *p*=0.044), having had STIs in the past year (aOR=2.3, 95% CI=1.0–5.0, *p*=0.038) and working as an MB (aOR=2.8, 95% CI=1.4–5.4, *p*=0.003). Age was not significantly associated with HIV prevalence.

**Table 4:** HIV prevalence and risk factors among repeat testers and first-time testers attending bathhouse in Tianjin, China

	Repeat testers (*n=*699)					First-time testers (*n=*943)				
		
		Univariate		Multivariate			Univariate		Multivariate	
						
Variable	HIV prevalence	OR (95% CI)	*P* value	OR (95% CI)	*P* value	HIV prevalence	OR (95% CI)	*P* value	OR (95% CI)	*P* value
Age(years)										
<25	12.8 (14/109)	1.0				8.1 (9/111)	1.0		1.0	
25–34	10.0 (27/269)	0.8 (0.4–1.4)	0.428			9.5 (27/283)	1.2 (0.5–2.6)	0.657	1.5 (0.7–3.5)	0.330
35–45	10.3 (21/203)	0.8 (0.4–1.6)	0.506			15.4 (54/350)	2.1 (1.0–4.3)	0.055	2.9 (1.3–6.4)	0.010
>45	13.6 (16/118)	1.1 (0.5–2.3)	0.874			17.6 (35/199)	2.4 (1.1–5.2)	0.025	3.6 (1.6–8.3)	0.003
Marital status										
Unmarried/divorced or widowed	11.9 (41/344)	1.0				13.5 (48/355)	1.0			
Married/cohabitated	10.4 (37/355)	0.9 (0.5–1.4)	0.530			13.1 (77/588)	1.0 (0.7–1.4)	0.852		
Residence										
Tianjin	7.7 (24/313)	1.0		1.0		12.0 (49/407)	1.0			
Other province	14.0 (54/386)	2.0 (1.2–3.2)	0.009	1.7 (1.0–2.9)	0.044	14.2 (76/536)	1.2 (0.8–1.8)	0.338		
Ethnicity										
Han Chinese	11.0 (75/681)	1.0				13.4 (123/919)	1.0			
Other	16.7 (3/18)	1.6 (0.5–5.7)	0.456			8.3 (2/24)	0.6 (0.1–2.5)	0.476		
Length of stay in Tianjin										
Less than 3 months	16.2 (31/191)	1.0				13.2 (35/266)	1.0			
Between 3 months and 2 years	9.5 (7/74)	0.5 (0.2–1.3)	0.163			10.2 (9/88)	0.8 (0.3–1.6)	0.471		
More than 2 years	9.2 (40/434)	0.5 (0.3–0.9)	0.012			13.8 (81/589)	1.1 (0.7–1.6)	0.814		
Educational Level										
Completed middle school	15.2 (24/158)	1.0				15.2 (41/269)	1.0			
High school or above	10.0 (54/541)	0.6 (0.4–1.0)	0.069			12.5 (84/674)	0.8 (0.5–1.2)	0.257		
Had STIs in the past year										
Yes	20.0 (9/45)	2.1 (1.0–4.6)	0.056	2.3 (1.0–5.0)	0.038	23.3 (7/30)	2.1 (0.9–4.9)	0.105		
No	10.6 (69/654))	1.0		1.0		12.9 (118/913)	1. 0			
Male sex worker (MB)										
Yes	25.4 (15/59)	3.1 (1.6–5.9)	0.001	2.8 (1.4–5.4)	0.003	34.5 (10/29)	3.7 (1.7–8.1)	0.001	6.3 (2.6–14.9)	<0.001
No	9.8 (13/640)	1.0		1.0		12.6 (115/914)	1.0		1.0	
AIDS knowledge										
Knowledgeable	11.4 (76/665)	1.0				13.0 (108/832)	1.0			
Not knowledgeable	5.9 (2/34)	0.5 (0.1–2.1)	0.327			15.3 (17/111)	1.2 (0.7–2.1)	0.496		
HIV risk awareness										
High	15.4 (6/39)	1.0				9.7 (3/31)	1.0			
Moderate	9.9 (23/232)	0.6 (0.2–1.6)	0.311			12.9 (49/379)	1.4 (0.4–4.7)	0.602		
Low or none	11.4 (49/428)	0.7 (0.3–1.8)	0.467			13.7 (73/533)	1.5 (0.4–5.0)	0.527		
Had anal intercourse (AI)										
Yes	11.0 (70/637)	1.0				13.6 (109/799)	1.0			
No	12.9 (8/62)	1.2 (0.5–2.6)	0.648			11.1 (16/144)	0.8 (0.5–1.4)	0.411		
Frequency of AI in last week										
≤2	10.3 (58/563)	1.0				12.9 (99/765)	1.0			
>2	14.7 (20/136)	1.5 (0.9–2.6)	0.145			14.6 (26/178)	1.2 (0.7–1.8)	0.555		
UAI with recent male sex partner										
Yes	12.1 (27/224)	1.0				14.7 (62/421)	1.0			
No	10.7 (51/475)	0.9 (0.5–1.4)	0.606			12.1 (63/522)	0.8 (0.5–1.2)	0.232		
UAI in the past 6 months										
Yes	11.0 (45/409)	1.0				14.6 (91/623)	1.0			
No	11.4 (33/290)	1.0 (0.8–1.3)	0.876			10.6 (34/320)	0.8 (0.7–1.0)	0.089		
Had group sex										
Yes	10.5 (9/86)	1.0				18.0 (18/100)	1.0			
No	11.3 (69/613)	1.1 (0.5–2.3)	0.827			12.7 (107/843)	0.7 (0.4–1.1)	0.141		
UAI in group sex										
Yes	10.5 (9/86)	1.0				23.3 (17/73)	1.0		1.0	
No	11.3 (69/613)	0.9 (0.6–1.3)	0.473			12.4 (108/870)	0.5 (0.3–0.9)	0.010	0.5 (0.3–0.9)	0.018
Had AI with commercial male sex partner(s)										
Yes	14.3 (7/49)	1.0				12.5 (9/72)	1.0			
No	10.9 (71/650)	0.8 (0.4–1.5)	0.511			13.3 (116/871)	1.1 (0.5–2.2)	0.844		
UAI in the last commercial male sex										
Yes	16.7 (3/18)	1.0				15.4 (4/26)	1.0			
No	11.0 (75/681)	0.6 (0.2–2.2)	0.456			13.2 (121/917)	0.8 (0.3–2.5)	0.746		
UAI in commercial male sex (past 6 months)										
Yes	13.2 (5/38)	1.0				18.9 (7/37)	1.0			
No	11.0 (73/661)	0.9 (0.6–1.5)	0.688			13.0 (118/906)	0.8 (0.5–1.2)	0.304		
Had sex with any female partner(s)										
Yes	8.7 (19/219)	1.0				13.1 (44/336)	1.0			
No	12.3 (59/480)	1.5 (0.9–2.5)	0.161			13.3 (81/607)	1.0 (0.7–1.5)	0.914		
Unprotected sexual intercourse with last female partner										
Yes	9.1 (13/143)	1.0				14.1 (37/262)	1.0			
No	11.7 (85/556)	1.3 (0.7–2.5)	0.380			12.9 (88/681)	0.9 (0.6–1.4)	0.627		
Unprotected sexual intercourse with any female partner (past 6 months)										
Yes	7.9 (13/164)	1.0				14.3 (42/294)	1.0			
No	12.1 (65/535)	1.3 (0.9–1.7)	0.136			12.8 (83/649)	0.9 (0.8–1.1)	0.530		

For first-time testers, MSM being over 45 years of age (OR=2.4, 95% CI=1.1–5.2, *p*=0.025) and MB (OR=3.7, 95% CI=1.7–8.1, *p*=0.001) remained at an increased likelihood of being HIV infected. Not having UAI during group sex was a protective factor (OR=0.5, 95% CI=0.3–0.9, *p*=0.010) (Table 4). In the multivariate model, being aged 35 to 45 years (aOR=2.9, 95% CI=1.3–6.4, *p=*0.010) and over 45 years (aOR=3.6, 95% CI=1.6–8.3, *p*=0.003), being a male sex worker (aOR=6.3, 95% CI=2.6–14.9, *p*<0.001) and not having UAI during group sex (aOR=0.5, 95% CI=0.3–0.9, *p*=0.018) remained significant (Table 4). There was a significant positive trend in HIV prevalence with age among first-time testers ($\chi_{trend}^{2}=9.816$, *p*=0.002), but no trend was detected across age groups among repeat testers ($\chi_{trend}^{2}=0.076$, *p*=0.783).

## Discussion

Our study confirmed that MSM repeat testers and first-time testers differed in HIV-related risk sexual behaviour and HIV prevalence. Almost half of the MSM attending the bathhouse VCT station had previously received HIV testing. HIV prevalence significantly increased with age among first-time MSM testers, but no differences were found among repeat testers. First-time testers over 25 years of age had 1.5 to 3.6 times HIV prevalence as those below 25 years. Among MSM older than 45 years in this group, HIV prevalence was found to be as high as 17.6%. These results corroborate findings from previous studies in China of increased HIV risk among older MSM [17, 18]. Data from a systematic review of HIV testing studies among Chinese MSM found evidence to support the relationship between age and repeat testing, where repeat testers tend to be younger men who visit MSM venues such as bathhouses more frequently [19]. Thus, younger MSM may have more access to venue-based HIV prevention interventions such as VCT. The difference in HIV prevalence by age in first-time testers but not in repeat testers suggests that introducing testing may detect more HIV cases in older MSM. Also, testing among all MSM should be promoted as a way to detect HIV cases earlier and decrease the transmission of HIV infection.

There was no difference in HIV prevalence between repeat testers and first-time testers. However, the context of HIV risk differed for repeat testers and first-time testers. Unknown serostatus persons were more likely than HIV-negative persons to engage in UAI with male partners, including during group sex and commercial sex. A higher proportion of first-time testers also reported practicing unsafe sex with their female partners. For first-time testers, VCT should be tailored to include basic HIV and AIDS knowledge as well as safer sex practices with both men and women. Given the high rate of both unprotected sex with female partners and marriage among first-time testers, comprehensive knowledge of safer sex practices is important to limit the transmission of HIV to otherwise low-risk female partners [20]. Our findings suggest that previous findings among Chinese MSM where married men were less likely to practice risk behaviours in order to protect their partners may not be applicable to this population [7].

In comparison, repeat testers were more likely than first-time testers to be not only knowledgeable about AIDS but also to have had an STI in the past year. Because risk behaviours for STIs are similar to those of HIV, it is likely that a substantial minority of MSM continue to engage in high-risk sexual behaviours [10]. As the risk of acquiring and transmitting HIV is amplified in the presences of STIs, it is necessary that post-test counselling addresses risk reduction for those chronically engaging in sexual risk behaviours [21].

This study also confirmed that male sex workers bore a disproportionate burden of HIV infection among both repeat and first-time testers. In this study population, MBs were three times more likely to be infected with HIV compared with non-sex workers. Among first-time testers, over one-third of all male sex workers were infected with HIV, and the risk of HIV infection for male sex workers was six times higher. These disparities are comparable to those reported in a study among MSM in Chengdu, China [22]. In our study, first-time MB testers were more likely to practice UAI during the past six months (82.8% vs. 54.2%; *χ*^2^=6.835, *p*=0.009). This result further supported the need to increase interventions targeting this group, including introducing VCT to MBs and increasing condom use not only with male clients but also with male casual sex partners and boyfriends [23, 24].

Over half of the patrons were non-local residents, among which almost half of the non-local residents had lived in Tianjin less than three years. Non-local residents were 1.6 times more likely to be HIV positive. Risk behaviours were more common among floating population than local residents because they were more likely to transmit HIV to people from/to other areas [25]. Health interventions which targeted the floating population should be carried out, such as rapid HIV screening.

There are limitations to the present study. First, this study was conducted in one MSM bathhouse, which may hinder generalizing our findings to the larger MSM population. Ethnographic data on MSM in China have found that sexual practices and risks vary across venues, so findings such as HIV risk from this bathhouse population may be substantially higher than lower risk venues of bars and teahouses [7, 26, 27]. Additionally, because our study was conducted at a venue rather than in the larger community, we were not able to reach MSM who did not frequent venues. However, given that widespread stigma against homosexuality is a significant barrier to participation in HIV prevention activities, it would have been difficult to conduct this study outside of a dedicated MSM venue [28]. Second, lack of response rate further undermines the generalizability of the study results. It is difficult to estimate how many and what proportion of the total number of customers has taken up the testing because hundreds of customers come in to the bathhouse every day and are constantly coming and going. Also, the fact that consent was collected might have influenced the composition of the sample. Men who did not want to be identified or documented as HIV positive may have been unwilling to take part in the study. However, we believe that the impact was minimal because all participants who were approached consented to participating in the study. Third, this study was cross-sectional, so causation cannot be established. Future longitudinal studies are needed to establish causation between repeat testing and changes in HIV risk. Finally, our study took a convenience sample. Snowball sampling might have influenced the ratio of previously tested because participants referred those they knew, who were likely to be quite similar to them. These results may not be applicable for MSM in regions with different HIV transmission dynamics or in rural areas.

## Conclusions

HIV prevalence differs significantly between repeat and first-time MSM testers. For MSM who have never been tested, VCT may be an important strategy to detect more HIV cases, especially among older MSM. For both repeat and first-time testers, VCT should be tailored to meet the specific needs of these groups. Given the higher proportion of repeat testers who are sex workers compared to first-time testers, VCT may be a viable channel to provide multiple opportunities for risk reduction consultations for this hidden population. Future studies should examine differences in HIV risk behaviours and social networks for older versus younger MSM, and how VCT should be implemented to reach subgroups such as older MSM to mitigate disparities within this population.

## References

[1] Ministry of Health of the People’s Republic of China (2012). 2012 China AIDS response progress report.

[2] Gao L, Zhang L, Jin Q (2009). Meta-analysis: prevalence of HIV infection and syphilis among MSM in China. Sex Transm Infect.

[3] Meng X, Zou H, Beck J, Xu Y, Zhang X, Miao X (2013). Trends in HIV prevalence among men who have sex with men in China 2003–09: a systematic review and meta-analysis. Sex Health.

[4] Zhang L, Gray RT, Wilson DP (2012). Modelling the epidemiological impact of scaling up HIV testing and antiretroviral treatment in China. Sex Health.

[5] Zou H, Hu N, Xin Q, Beck J (2012). HIV testing among men who have sex with men in China: a systematic review and meta-analysis. AIDS Behav.

[6] Song Y, Li X, Zhang L, Fang X, Lin X, Liu Y (2011). HIV-testing behavior among young migrant men who have sex with men (MSM) in Beijing, China. AIDS Care.

[7] Wei C, Ruan S, Zhao J, Yang H, Zhu Y, Raymond HF (2011). Which Chinese men who have sex with men miss out on HIV testing?. Sex Transm Infect.

[8] Li X, Lu H, Raymond HF, Sun Y, Jia Y, He X (2012). Untested and undiagnosed: barriers to HIV testing among men who have sex with men, Beijing, China. Sex Transm Infect.

[9] Choi K, Lui H, Guo Y (2006). Lack of HIV testing and awareness of HIV infection among men who have sex with men, Beijing, China. AIDS Educ Prev.

[10] Matovu JKB, Gray RH, Kiwanuka N, Kigozi G, Wabwire-Mangen F, Nalugoda F (2007). Repeat voluntary HIV counseling and testing (VCT), sexual risk behavior and HIV incidence in Rakai, Uganda. AIDS Behav.

[11] Fernández MI, Perrino T, Bowen GS, Royal S, Varga L (2003). Repeat HIV testing among Hispanic men who have sex with men – a sign of risk, prevention, or reassurance?. AIDS Educ Prev.

[12] Leaity S, Sherr L, Wells H, Evans A (2000). Repeat HIV testing: high-risk behaviour or risk reduction strategy?. AIDS.

[13] MacKellar D, Valleroy L (2002). Repeat HIV testing, risk behaviors, and HIV seroconversion among young men who have sex with men: a call to monitor and improve the practice of prevention. J Acquir Immune Defic Syndr.

[14] Huang ZJ, He N, Nehl EJ, Zheng T, Smith BD, Zhang J (2012). Social network and other correlates of HIV testing: findings from male sex workers and other MSM in Shanghai, China. AIDS Behav.

[15] Fisher J, DelGado B (2002). The dynamics of repeat HIV testing, and interventions for repeat HIV testers. AIDS Behav.

[16] Bao Y, Zhang Y, Zhao J, Sun J, Tan H (2009). [HIV infection and KAP status among men who have sex with men in 14 Chinese cities]. Zhonghua Yu Fang Yi Xue Za Zhi.

[17] Feng L, Ding X, Lu R, Liu J (2009). High HIV prevalence detected in 2006 and 2007 among men who have sex with men in China’s largest municipality: an alarming epidemic in Chongqing, China. J Acquir Immune Defic Syndr.

[18] Xiao Y, Sun J, Li C, Lu F (2010). Prevalence and correlates of HIV and syphilis infections among men who have sex with men in seven provinces in China with historically low HIV prevalence. J Acquir Immune Defic Syndr.

[19] Chow E, Wilson D, Zhang L (2012). The rate of HIV testing is increasing among men who have sex with men in China. HIV Med.

[20] Parish WL (2003). Population-based study of chlamydial infection in China: a hidden epidemic. JAMA.

[21] Fleming DT, Wasserheit JN (1999). From epidemiological synergy to public health policy and practice: the contribution of other sexually transmitted diseases to sexual transmission of HIV infection. Sex Transm Infect.

[22] Feng Y, Wu Z, Detels R, Qin G (2010). HIV/STD prevalence among MSM in Chengdu, China and associated risk factors for HIV infection. J Acquir Immune Defic Syndr.

[23] Liu S, Zhao J, Rou K, Chen L, Cai W, Li L (2012). A survey of condom use behaviors and HIV/STI prevalence among venue-based money boys in Shenzhen, China. AIDS Behav.

[24] Liu S, Chen L, Li L, Zhao J, Cai W, Rou K (2012). Condom use with various types of sex partners by money boys in China[J]. AIDS Educ Prev.

[25] Shi TX, Zhang BC, Li XF, Xu JX, Wang N, Yu ZZ (2009). Study on the high risk behaviors related to AIDS among men who having sex with men in the floating population. Zhonghua Liu Xing Bing Xue Za Zhi.

[26] Choi K, Diehl E, Yaqi G, Qu S, Mandel J (2002). High HIV risk but inadequate prevention services for men in China who have sex with men: an ethnographic study. AIDS Behav.

[27] Li H, Lau JTF, Holroyd E, Yi H (2010). Sociocultural facilitators and barriers to condom use during anal sex among men who have sex with men in Guangzhou, China: an ethnographic study. AIDS Care.

[28] Feng Y, Wu Z, Detels R (2010). Evolution of MSM community and experienced stigma among MSM in Chengdu, China. J Acquir Immune Defic Syndr.

